# Drug-Resistant Bacterial Infections and Alternative Therapies

**DOI:** 10.3390/biomedicines13020457

**Published:** 2025-02-13

**Authors:** Vijay Singh Gondil

**Affiliations:** Department of Microbiology and Immunology, University of Rochester Medical Center, Rochester, NY 14642, USA; vjgondal@gmail.com

The introduction of antibiotics in clinical use has revolutionized modern medicine practices and significantly contributed to the control of bacterial infections, which were the leading cause of morbidity and mortality throughout human history. The mid-twentieth century (1930–1960) was the period when the discovery and development of antibiotics reached its peak, often referred to as the “Golden Era of Antibiotics”. With their development, effectiveness, and popularization, antibiotics have been overused and misused in healthcare, animal husbandry, and agriculture, leading to the development of antibiotic resistance in bacterial populations. Antibiotic resistance is one of the major healthcare concerns, as many modern medicine practices, including organ transplants, surgeries, and cancer treatment, would be impossible without the existence of effective antibiotics [1,2]. Unfortunately, the dramatic global increase in antibiotic-resistant pathogens to available antibacterial agents cannot be countered by the current slow pace of antibiotic development [3]. Antibacterial development progresses much slower than the development of drugs for chronic diseases. For instance, while there are around 4,000 immuno-oncology drugs, only 30–40 antibacterial molecules targeting World Health Organization priority pathogens are currently in clinical development [4]. The gap in antibiotic discovery has driven researchers to study the mechanisms of antibiotic resistance and alternative therapies to antibiotics for the treatment of drug-resistant bacterial infections. Considering these facts, we have created the Special Issue “Drug-Resistant Bacterial Infections and Alternative Therapies” for MDPI’s *Biomedicines*. This Special Issue will feature review and research articles that highlight the critical issue of drug-resistant infections and the potential of alternative therapies in combatting drug-resistant bacterial infections, particularly as conventional antibiotic treatments become increasingly limited due to the emergence of antibiotic resistance. 

Starting with the application of green synthesis of silver nanoparticles against muti-drug-resistant bacterial infections in aquaculture, Raza et al. analyzed the green synthesis of nanoparticles using leaf extract from *Mangifera indica.* These silver nanoparticles were characterized using various physical methods and further explored as an alternative therapeutic agent to treat *Aeromonas hydrophila* infection in the *Cirrhinus mrigala* model. These green-synthesized nanoparticles demonstrated a reduction in bacterial burden for *A. hydrophila* in the *C. mrigala* model, with the recovery of liver enzymes, reduced oxidative stress, and improved tissue histology compared to the control groups. This study provides valuable insight into alternative therapeutic agents for the treatment of bacterial infection in aquaculture. In another study, Streltsova et al. presented alterations in renal pelvis function and morphology following antibacterial photodynamic therapy. In this study, a photosensitizer (photoditazine) was administrated to the kidneys of animals and excited to induce the effects of photodynamic therapy. The results showed that the fluorescence of the photosensitizer was not detected in the urothelium of the renal pelvis, and minor changes were observed in the urothelium of the renal pelvis, with no impact on the underlying connective tissue. Moreover, no alterations in renal function were recorded after the treatment, suggesting that antibacterial photodynamic therapy can be used with available antibiotic therapies in renal surgical procedures. 

Further, Chen et al. evaluated the synergistic antibacterial effects of common nonsteroidal anti-inflammatory drugs and standard-of-care antibiotic treatment against several cystic fibrosis pathogens. The results showed that a high dose of ibuprofen exhibited antimicrobial activity and demonstrated synergistic antibacterial activity with ceftazidime against cystic fibrosis clinical isolates. Moreover, the *Pseudomonas aeruginosa-induced* pneumonia murine model treated with a combination of ceftazidime and ibuprofen showed higher survival rates in animals compared to animals treated with a single drug (ibuprofen or ceftazidime) and control animal, showing the potency of NSAIDs in combination with standard-of-care antibiotics in an animal infection model. To understand the biology and drug-resistant mechanisms of *P. aeruginosa*, Suh et al. investigated the outer membrane vesicles of *P. aeruginosa* and their inhibitory effect on *Acinetobacter baumannii*. Outer membrane vesicles from different *P. aeruginosa* strains were extracted, characterized, and compared; outer membrane vesicles from strain PA022 showed significant antibacterial activity against *A. baumannii* in cross-streak and time-kill assays compared to outer membrane vesicles from PA ATCC 27853. The extracted vesicles were characterized by transmission electron microscopy, SDS-PAGE, nanoparticle tracking, and proteomics analysis. Proteomic analysis recognized 623 and 538 proteins in PA022 and PA ATCC 27853 outer membrane vesicles, respectively. Furthermore, six virulence factors and mobility-associated proteins were also characterized from PA022 outer membrane vesicles. The diversity and higher abundance of proteins in the outer membrane vesicles are promising for investigating bacterial interactions and other signaling pathways in future studies. 

Further, a review article by Sabo et al. provided an overview of significant developments in managing infections in critical care facilities based on six randomized trials published in the year 2023. This review focused on the outcome of randomized clinical trials related to infection prevention, current standard-of-care antibiotic regimes, and antibiotic adjunct treatments. This article emphasizes a multidisciplinary approach to applying clinical evidence to reduce bacterial infections and improve clinical outcomes in critically ill patients. Additionally, in a related context, Negi et al. reported the antibacterial and antibiofilm activities of green-synthesized graphene silver nanoparticles against multidrug-resistant nosocomial pathogens. In this study, green synthesis and characterization of graphene silver nanoparticles were performed, followed by antibacterial, antifungal, and antibiofilm assays. The synthesized nanoparticles were found to be antibacterial and antifungal, showing biofilm inhibition of up to 80–96% against various fungal and bacterial pathogens. The graphene silver nanoparticles were also impregnated onto urinary catheters to evaluate cellular adhesion and biofilm formation; these urinary catheters functionalized by graphene silver nanoparticles prevented biofilm formation by *Candida auris* and *P. aeruginosa*. Graphene silver nanoparticles also exhibited hemocompatibility and biocompatibility, demonstrating that they are promising candidates for animal studies and further therapeutic development. 

Lastly, this Special Issue concludes with an interesting study by Islam et al., which reported a novel *Escherichia coli* O157:H7 phage, SPEC13, as an alternative therapeutic agent to counter food-borne *E. coli* infections. In this study, a novel bacteriophage was isolated from a sewage sample, showing 100% lytic potential against tested O157:H7 and non-O157:H7 *E. coli* bacteria. Genome studies revealed that the bacteriophage SPEC13 belongs to an unknown genus and lacks a lysogenic cycle and antibiotic-resistant genes, positioning it as a promising alternative therapeutic agent. Along with in vitro assays, the bacteriophage SPEC13 also showed its effectiveness in the murine infection model by significantly reducing bacterial burden, edema, and tissue damage. 

Overall, we believe that the collection of articles in this Special Issue will not only assist scientific communities and clinicians in understanding the emerging antibiotic resistance crisis and the gap in drug discovery void but also guide the development of a newer generation of alternative therapeutic agents. By highlighting these critical areas, we aim to accelerate the ongoing advancements in combatting drug-resistant infections and foster better patient outcomes on a global scale.

## Abbreviations

NSAIDs: Nonsteroidal Anti-Inflammatory Drugs, ATCC; American Type Culture Collection, SDS-PAGE; Sodium Dodecyl Sulfate Polyacrylamide Gel Electrophoresis.
